# Outreach simulation for system improvement: a novel advocacy and reporting process

**DOI:** 10.1186/s41077-025-00372-0

**Published:** 2025-09-01

**Authors:** A. St-Onge-St-Hilaire, B. Lawton, L. Dodson, J. Acworth, D. Hufton, B. Symon

**Affiliations:** 1 Simulation Training Optimising Resuscitation for Kids (STORK) Statewide Simulation Service, Queensland Children’s Hospital, South Brisbane, QLD Australia; 2 School of Medicine & Dentistry, Griffith University, Brisbane, Australia; 3 Faculty of Medicine, University of Queensland, Brisbane, QLD Australia; 4 NHS Forth Valley, Scottish Centre for Simulation and Clinical Human Factors (SCSCHF), Larbert, Scotland

**Keywords:** Translational simulation, Outreach simulation, Quality improvement, System-level change, Latent safety threats, In situ simulation, Healthcare systems improvement, Simulation reporting, Kirkpatrick model, Pediatric resuscitation, Simulation impact measurement, Simulation-based education, Regional healthcare, Human factors in simulation

## Abstract

**Introduction:**

Healthcare simulation programmes measuring their value risk wasting resources in attempts to prove they impact patient outcomes. Simulation is one of many strategies used to enhance healthcare systems, and proving specific correlation with simulation will prove impossible in many circumstances. To maintain accountability but ensure feasibility, we argue simulation services need measurement processes that are robust, achievable, and synergistic with their mission. In 2023, the STORK service in Queensland, Australia, began measuring the impact of simulation on *systems* rather than *patients* to define the extent to which their educational programmes could impact system improvement.

**Methods:**

Translational simulation methodologies and quality improvement measures were embedded in an established educational course. We used simulation activities to diagnose environmental and system-level problems in participants’ workplaces throughout Queensland. Courses included dedicated time to discuss site-specific actionable solutions with participants and identified local champions to implement quality improvement changes. By designing a novel electronic reporting process (Optimus PRIME Course Summary), we documented issues and solutions identified in regional healthcare facilities and ensured they reached key stakeholders. We audited our ability to improve these systems through follow-up data collection via phone and emails with local educators across the state.

**Results:**

From 40 courses delivered across 37 facilities, 242 issues were identified, primarily related to drug safety and equipment management. At follow-up, 45.5% of the issues were resolved, with 44.6% still being addressed. Recommended resources were successfully implemented in 64% of sites.

**Conclusion:**

This process demonstrates that focusing on system-level changes can significantly enhance healthcare systems. The reporting framework provided a robust, achievable, and synergistic method to measure simulation impact and influence change. Additionally, we share key lessons learned from the process to guide other simulation services in improving their own measurement strategies.

**Graphical Abstract:**

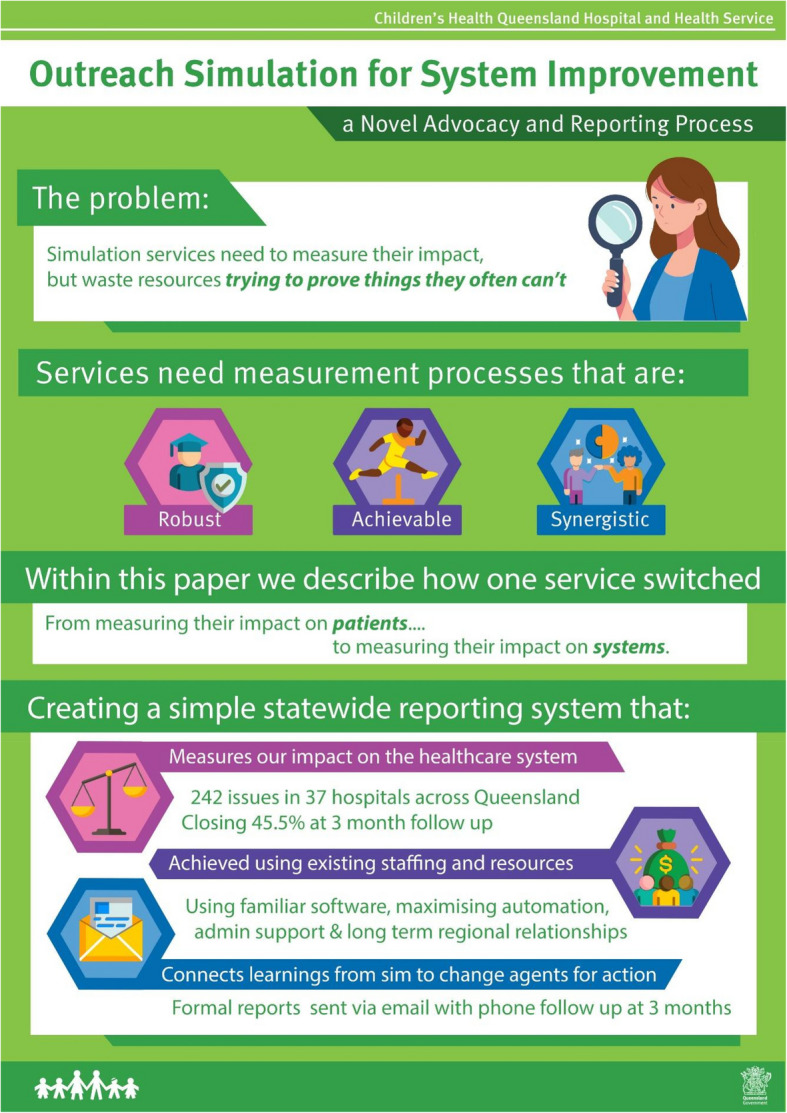

**Supplementary Information:**

The online version contains supplementary material available at 10.1186/s41077-025-00372-0.

## Introduction

Simulation programmes face extraordinary challenges when trying to measure their clinical impact. Firstly, the research processes required are confounded by the scope and complexities of modern healthcare systems. Secondly, meaningful research of these systems demands resources that can exceed service capacity. Thirdly, the research process itself can ironically distract from service delivery and fulfilment of the mission of the service. While it is imperative for simulation services to assess their impact, we lack what is needed: robust, achievable, and synergistic processes to do so.

An academically robust approach is essential for simulation service data to be meaningful. Many frameworks to facilitate measurement of clinical impact exist, including Kirkpatrick’s model of training evaluation [1–3]. Kirkpatrick presents a hierarchical model involving four levels of evidence: reactions, learning, behaviour, and results. While this is an approachable framework for discrete interventions, it is not so easily applied to complex healthcare systems. Hospitals are not petri dishes designed for the discrete application and measurement of individual interventions; they are vast in scale and complexity. In a workplace both complicated and complex, determining what intervention is truly responsible for any particular outcome is challenging. This challenging task can become herculean when teams seek to correlate ‘simulation interventions’ directly with ‘patient outcomes’. Varpio and Sherbino argue that direct correlation of education with patient outcomes is a ‘largely unattainable goal’ [4], and we propose simulation is often an excellent example. Services pursuing unrealistic measurement goals risk wasting resources in pursuit of evidence that is unlikely to be proven and unhelpful for their cause. This does not mean measurement is futile, but rather that services should select robust measurement metrics that can actually lead to meaningful conclusions. Avoiding measuring impact altogether sacrifices opportunities for accountability, reflexivity, and service improvement. We highlight that measuring our impact matters, but our measurement processes need to be synergistic with our service’s mission.

Despite an abundance of simulation research around programmatic evaluation [5–8], current literature lacks sufficient industry-focused examples of real-world assessment of large educational simulation services. This gap is a lost opportunity to report on improvement processes for the simulation community as a whole. Well-described examples contribute to the simulation community by providing advocacy for meaningful measurement metrics, concrete examples for exploration, critique and consideration by other teams, and reflections and learnings to influence the planning of other teams along their assessment journeys.

With this research gap in mind, we will describe how our simulation outreach service ‘STORK’ embedded a reporting system into our paediatric simulation curriculum in 2023. Recognising that proving our education’s influence on real paediatric resuscitations was unrealistic, we pivoted instead to measuring our impact on resuscitation *systems.* The reporting system allowed us to draw robust conclusions about our influence on paediatric resuscitation systems, was achieved with no additional staffing or funding, and furthered our mission to improve the resuscitation of sick children doubling as an advocacy tool for organisational change.

We describe our educational strategy, our reporting and follow-up processes, our impact on clinical systems, and lessons learned from the first year. In doing so, we aim to empower other simulation outreach services to connect their education more intentionally to quality improvement processes.

## Background

### The STORK service

Founded in 2014, the Simulation Training Optimising Resuscitation for Kids (STORK) team from Children’s Health Queensland delivers courses to clinicians across Queensland, Australia. Our programme’s mission is'ensuring every child in Queensland has access to optimal resuscitative care’. During our first decade we pursued this mission through extensive provision of resuscitation education, delivering 100–140 courses per year, to over 60 hospitals. This involved every Hospital and Health Service in a state more than twice the size of Texas, USA.

The STORK team comprises four part-time paediatric emergency medicine physicians, two part-time simulation fellows, two nurse educators, five part-time simulation coordinators, and one administration officer.

The courses we deliver are structured around a spiral curriculum named OPTIMUS (Optimising Paediatric Training In Emergencies Using Simulation). The curriculum consists of four major constituent courses targeted as either skills development or skills maintenance vehicles for first or second tier responders. Additional simulation packages offer spaced repetition opportunities. Faculty resources are shared online for non-commercial use, with courses delivered by a combination of STORK staff and regional educators.

Each simulation coordinator is responsible for 10–12 sites and for communication regarding courses, site summaries, and follow-ups. This model has helped our team build and maintain relationships with colleagues working in rural and remote sites, and over time has generated buy-in for our service.

### Changing our approach to measurement

As a simulation education service, we wanted to understand the impact of our work and to advocate for meaningful improvements to the healthcare system. Measuring impact on actual paediatric resuscitations was desirable but not feasible. Any improvement in statewide paediatric outcomes would be tempting to claim but statistically dubious. Our previous attempts to monitor our performance included low-level Kirkpatrick data: Staff surveys (which were highly positive) and narrative feedback (that our service influenced subsequent resuscitations). While that data was appreciative, it was not constructive, furthering neither refinement of our work nor understanding of our impact. We needed a robust but achievable alternative that did not cost additional resources.

Supported by recurrent funding from our organisation, our motivation to measure impact was intrinsically driven. We had noticed recurrent resuscitation challenges when returning to the same hospitals and shared concern about the limits of education’s impact on healthcare. Our simulations provided rich data about barriers to optimal care in regional settings, but these insights were not consistently translated into action. We recognised a ‘powerful debrief’ was not enough. Local teams needed the information we were finding synthesised and reported on. Accepting that ‘education was essential, but insufficient’ [9] prompted us to embed quality improvement concepts within our second generation courses and pivot from hypothesising impact on resuscitated *patients* to measuring impact on resuscitation *systems*.

To keep our methods *robust*, we began to document, report, and follow up on every latent safety threat identified on our courses. To keep the process *achievable*, we utilised familiar technology: a combination of sticky notes, Microsoft Forms, and Office. To keep our process *synergistic* with our mission, we designed our reports for internal and external use, sharing with local hospitals. In doing so, we created a process that served as an internal auditing tool for us and an external advocacy tool for regional change agents. In our first year, we documented and published changes in regional sites that had previously proved elusive [10].

In our previous, related publication in an Australian clinical journal, we have described our reporting process, hospital demographics, our data analysis methods, and the recurrent themes we identified. Within this current paper, written for a simulation-focused readership, we attempt to highlight more specifically our pedagogical choice to deliberately merge educational experiences with quality improvement moments. We describe our course delivery structure, our assessment of the service within Kirkpatrick’s framework, and lessons learned for other services seeking to embed quality improvement within their education.

## Methods

### Incorporating quality improvement into the Optimus PRIME course

Our most frequently delivered course, ‘Preparing for Retrieval In Medical Emergencies (Optimus PRIME)’ focuses on resuscitation and retrieval of critically unwell children. We support participants to build on their current knowledge with an intentionally constructivist approach to learning. We focus on *how* to perform recurrent skills and *why* non-recurrent skills are performed in a particular way [11]. Independent knowledge and skills application is supported during immersive simulation exercises.

**Fig. 1:**
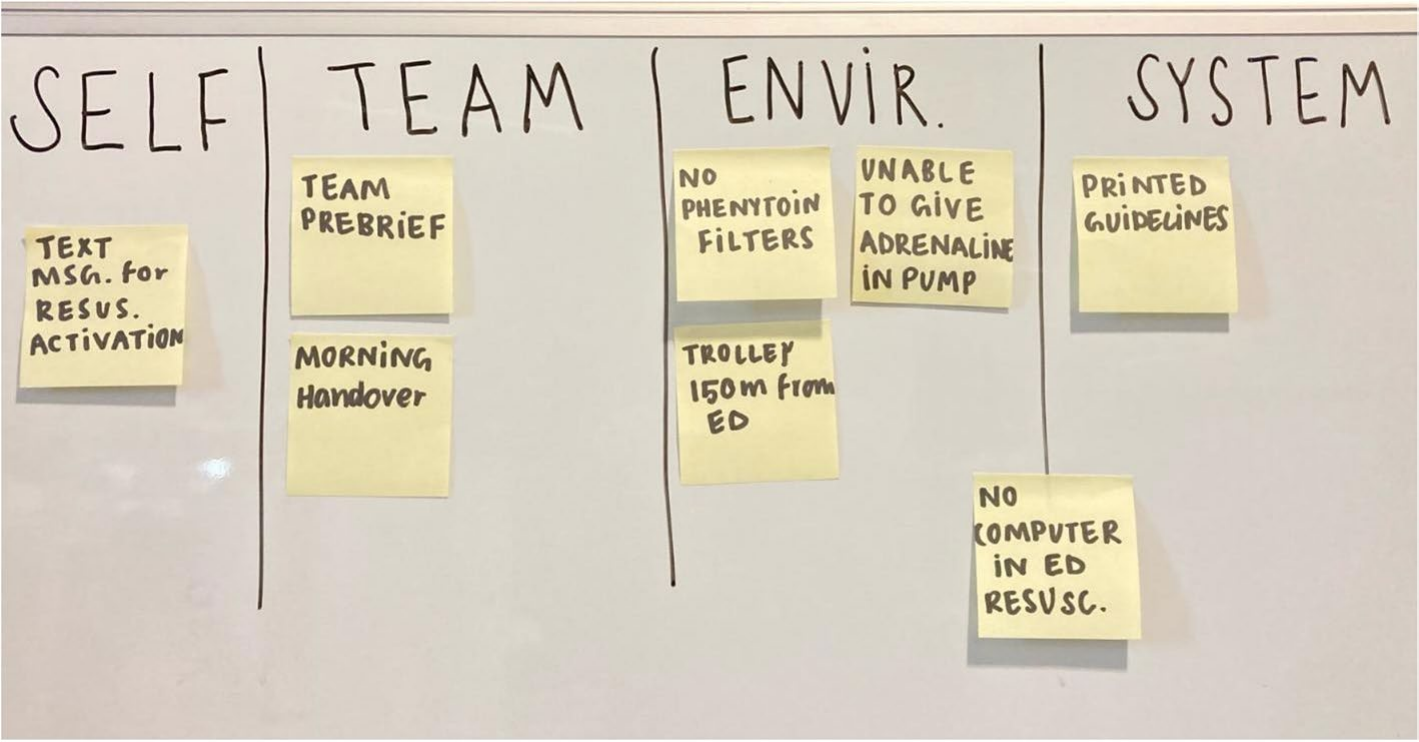
Visual display of sticky notes categorised using the ‘Self, Team, Environment, System’ framework

In our second iteration of the course, we introduced principles from translational simulation and quality improvement science [12]. The 4-level Human Factor Framework ‘Self, Team, Environment and System’ by Hicks and Petrosoniak [13] is described to participants early in the day. Faculty encourage participants to identify issues within each level during the course, encouraging reflection on ways to improve care at each level. Issues are documented on sticky notes and displayed (Fig. 1). During course closure, participants and faculty review all sticky notes to form consensus, identify solutions, and nominate local stakeholders to champion them. Local champions are staff with influence on paediatric healthcare delivery, commonly nurse educators, nurse unit managers, pharmacists, and medical staff with paediatric portfolios. Observations, suggestions, and contact details are recorded via Microsoft Forms.

### Optimus PRIME site summary design

The Optimus PRIME Site Summary is a 2-page report divided into four sections: (1) demographics (site name, date of visit, course delivered), (2) service strengths, (3) issue categories including challenges, suggested actions, and stakeholder contact for each, and (4) additional resources. A standardised appendix contains information on equipment ordering codes, their relevance in resuscitation, and suggestions for additional training or resources. Feedback is focused on actionable, contextualised solutions, with an emphasis on co-generation and ownership by local course participants [see Supplementary material 1: Optimus PRIME site summary].

The initial design phase included meetings to identify key reporting categories. Existing systems for categorising latent safety threats were considered, such as SEIPS and FMEA analysis [1, 14]; however, we believed a report containing strict risk categorisations could be perceived as external policing of local practice and damage rapport. Instead, our team elected for a course-specific ‘site summary’ form developed internally. To enhance adoption, we have deliberately designed the report to be aesthetically approachable, using simple language and practical suggestions. Five categories were identified as aligning well with the Human Factor framework: Strengths, Teamwork, Drug Safety, Equipment, and Department Layout and Resources. Selection was informed by faculty knowledge about common issues identified on courses, as well as published work on paediatric readiness [15].

A data collection form was created in *Microsoft Forms* using a mix of closed and forced-choice answers to facilitate the collection of demographic data and free text. Form submission auto-generates a *Microsoft Word* document within *Microsoft Teams*, importing information into a site summary template.

Several design decisions were embedded within the report structure: it’s colourful and visually appealing, strengths are featured early to build rapport and reflect a Safety 2 mindset that explores the components of success [16], and recommendations are action-oriented. We highlight that the summary synthesises challenges and solutions identified on the day, and is not an independent measure of service quality. Participant performance is not measured.

The report system was created with an iterative design cycle approach. Over the first year, it was repeatedly adapted in response to feedback. For example, the term ‘PRIME Report’ was changed to ‘PRIME Site Summary’ in response to individual feedback regarding perceived evaluative stress.

### Optimus PRIME site summary delivery and follow-up

After each course, a simulation coordinator revises the auto-generated summary document, refining wording and ensuring accuracy. A PDF copy is emailed to stakeholders identified on the course. A pre-scripted email introduction emphasises the summary is offered in good faith and not intended as an assessment. We target distribution of the summary within 14 days, and on a case-by-case basis our simulation coordinators often send additional emails to specific local stakeholders to help kick-start conversations about improvement opportunities.

A 3-month follow-up is organised with each site to review interval changes made within their system and explore ongoing challenges. Agenda items from the reports are discussed and categorised as either ‘no further action required', ‘work in progress’ or ‘item closed without action’ and documented within a Microsoft Excel spreadsheet. Stakeholders are encouraged to provide feedback about the process itself.

### Ethics

This project was granted an ethics review exemption from the Children’s Health Queensland Human Research Ethics Committee as a Quality & Safety Project. No data on individual performance was collected. Data was collected electronically on a secure server, resulting in no paper trace, and extracted to the abstraction form with access limited to the investigators only.

### Data collection and analysis

Data from site summaries and follow-ups were extracted by a researcher [A.SOSH] using a locally created standard abstraction form. Common themes were extracted, focusing on systemic issues or those representing latent safety threats. Descriptive statistics for categorical variables were calculated using proportions. Non-parametric tests were used due to the wide distribution of the data and presence of outliers. We have previously published the prominent latent safety threats identified, including a more detailed description of our research methodology [10].

## Results

Between March and December 2023, 40 Optimus PRIME courses were delivered in 37 healthcare facilities. Our faculty engaged with 346 participants; 72% were nurses and 28% doctors or medical trainees. Thirty-nine site summaries were distributed, as one site was visited a second time before the summary completion.

A total of 242 problems were diagnosed during our study period. A median of 6 issues were discovered by participants per site, with problems relating to drug safety and equipment being more frequent (Table 1).

**Table 1:** Findings of the initial Optimus PRIME report summaries

Initial Optimus PRIME report summary		
Issues per category	Count (Mean per site; SD)	Common themes
Drug safety	81 (2.1; 1)	Infrequent infusion pump safety software library updates
Equipment	82 (2.1; 1.64)	Challenges with consistently maintaining paediatric airway equipment Lack of internet and computer access in ED
Resources	48 (1.23; 0.71)	Challenges with access and awareness of statewide open access resources
Teamwork	31 (0.79; 0.89)	Highly valued rich close knit relationship with colleagues Frequent hospital staff turn over
Total	242 (6.2; 2.9)	

At site follow-up, 45.5% of issues were resolved while 44.6% were still being addressed (Table 2). Most sites (64%, *n* = 25) were able to order resources recommended.

**Table 2:** Findings of the follow-up process of the Optimus PRIME reports summaries

Follow-up of Optimus PRIME summary	
Overall quality improvement metrics	Count (percentage)
Improvement from initial summary	218/242 (90%)
• Issues resolved	110/242 (45.5%)
• Issues as work in progress	108/242 (44.6%)
• Issues with no action undertaken	18/242 (7.4%)
• Data not available	6/242 (2.5%)
Sites able to order new resources	25/39 (64%)

With respect to our performance, delays were noted with both the initial summary delivery (Median (IQR) 40 days [19.75–53.25]) and the timing of follow-ups (Median (IQR) 161 days [130.5–232.5]). The follow-up process took an average of 47 min per course to complete.

## Discussion

The following discussion explores how our approach evolved to meet criteria of robustness, achievability, and synergy with our mission. Our site summary process aimed to create a *robust* method of measuring the impact of our simulation programme, that was *achievable* with our current staffing and *synergistic* with our organisational mission. Within our discussion, we reflect on each of these goals in turn.

### A more robust method of measuring the impact of a simulation programme

Simulation services seeking to measure their impact often reference Kirkpatrick’s framework [2]. Kirkpatrick presents a hierarchy of four levels of measures, moving through participant reactions (e.g. participant surveys on engagement and relevance), learning (e.g. measured improvement on the day), behaviour (e.g. integration of new skills into actual workplace practice) and results (e.g. patient outcomes and organisational changes that occur subsequent to training).

We had previously attempted to evaluate our training to the Kirkpatrick ‘Reactions’ level with participant surveys. Feedback and our net promoter score showed that we were a popular service, but comments were mostly appreciative rather than constructive.

We intentionally avoid summative assessment of ‘Learning’ in our PRIME course due to concerns of generating high levels of social-evaluative stress when visiting regional areas. We argued that ‘Learning’ was partially demonstrated when individuals shared take-home learnings at the end of the course.

**Fig. 2:**
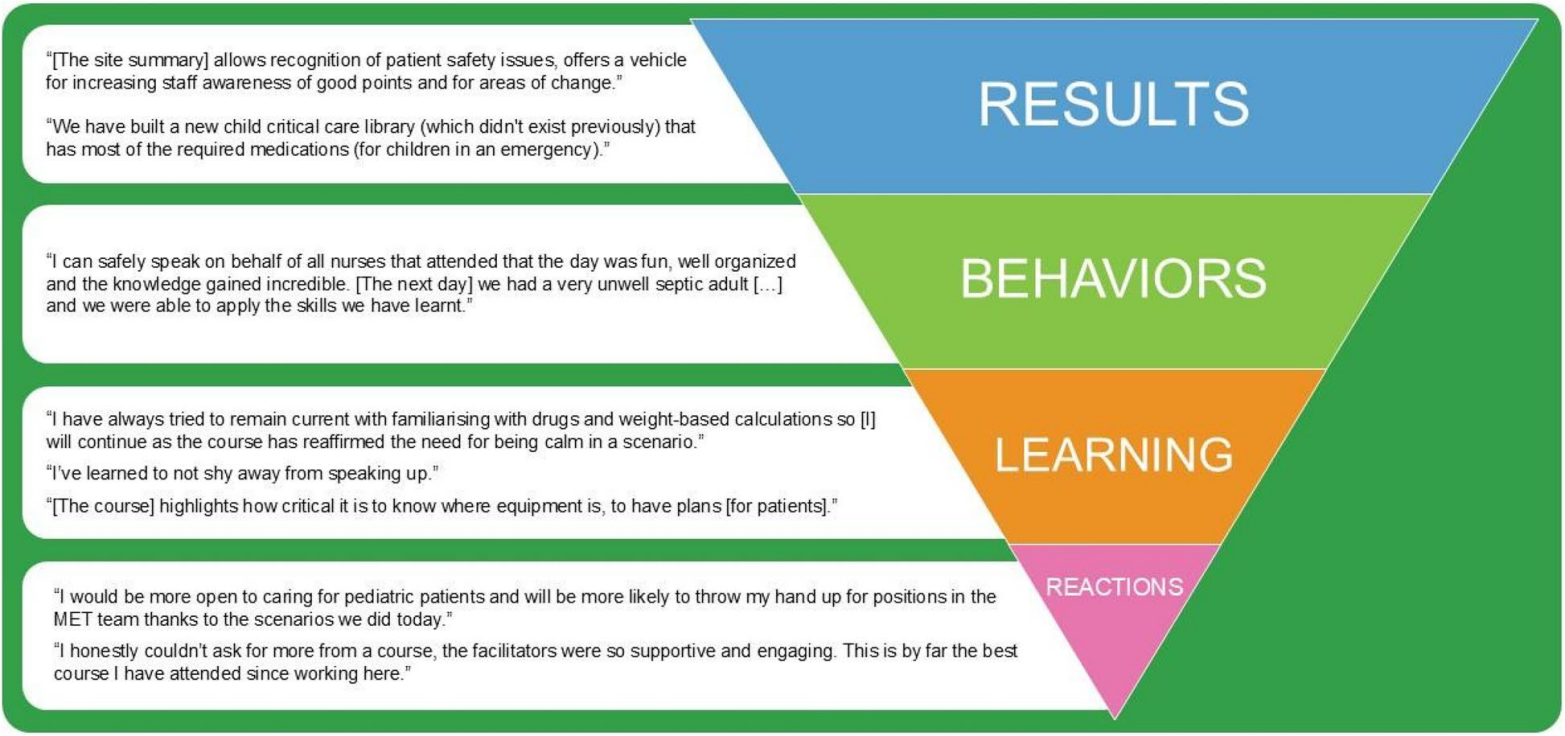
Narrative comments and feedback from course participants and educators in relation to Kirkpatrick’s model

The effectiveness of our course at the ‘Behaviours’ level was reflected in participants’ emails recounting successful resuscitations after the course (Fig. 2). These accounts illustrate changes at Kirkpatrick’s Level 3, showing that participants adopted behaviours capable of influencing and shaping an established culture. Our approach incorporated specific learning principles and practical design choices to drive these behavioural outcomes. Drawing from social cognitive learning principles, we structured simulations in situ to create an authentic, engaging representation of ‘work as done’ rather than ‘work as imagined’. This approach mirrors real-world practices, providing concrete experiences essential to enhance learning, trigger reactions, and shape behaviours. By using authentic equipment and forming realistic teams with their inherent dynamics—while fostering problem-solving skills and generating practical, context-specific solutions—we empowered participants to adopt and sustain behaviours that would directly impact their clinical practice.

While these narratives enriched our understanding of our impact, we lacked comprehensive data to confirm widespread impact and lacked a measure to assess the ‘Results’ level. By shifting our focus from patient to system improvement, we documented site-specific work against identified issues, confirming discrete episodes of organisational change consistent with Kirkpatrick’s ‘Results’. Importantly, we do not pretend these successes are ours alone, but argue they are the result of collective and coordinated action between simulation outreach and local clinicians. Our follow-up process documented organisational, equipment, and process changes at individual hospitals (Table 2). With 45% of issues fully resolved within 3 months and an additional 45% in progress, we documented changes on all 4 levels of Kirkpatrick’s model. We also celebrated and propagated regional expertise and innovations across the state. These results highlight the strength of our reporting system, particularly its ability to harness individual clinicians’ capacity to champion, coordinate, and generate organisational changes.

### An achievable method of measuring the impact of a simulation programme

Our reporting process was facilitated without additional staffing or expenses. This was achieved through the use of familiar software, maximising automation, administrative support, and longitudinal relationships with regional sites.

We utilised Office 365 software throughout the process. Staff were familiar with it, and the integration of QR codes to link to reporting forms enabled staff to quickly complete data collection on their smartphones during course closure or travel time from courses. Familiar technology avoided adding further barriers to data collection.

A combination of software automation and consistent administrative support was essential. Forms and templates were co-designed by our administrative officer, ensuring every process was automated as much as possible. The use of *Microsoft Forms* to auto-generate a *Microsoft Word* document decreased report writing times. Our administrative officer created automatic email reminders to prompt timely site summary completions.

In addition to the technical processes described, we believe our new process was successful because it leveraged relationships built through a decade of delivering paediatric education to every hospital and health service in Queensland. While this education provided individual clinicians with essential training, it also fostered our relationship with other clinical teams. Outreach education has been described as a relational intervention [17, 18], and as such, we regard our courses as vital steps in building psychologically safe relationships with regional educators and clinicians. Had we rolled out a statewide reporting process on paediatric resuscitation at the start of our 10-year journey, we believe we would have decimated an early regional courtship. As such, we strongly recommend other outreach services build psychological safety and establish relationships before launching into this depth of reporting.

While we believe success was informed by these deliberate choices (familiar software, maximising automation, administrative support, and longitudinal relationships), the process had challenges. Report writing was an unappealing new experience for many in our team, and we faced difficulties meeting the pre-established contact points of 14 days and 3 months. Follow up was made more difficult due to frequent regional personnel turnover in educational and management roles. This caused interruption in communications and challenges coordinating time with educators amidst clinical duties. Some coordinators reported frustration with the additional taskwork, although this decreased with familiarity and time. Additional support processes were arranged for report completion. Two senior clinicians (BS and JA) with confidence writing diplomatic, unambiguous communication took on a ‘reviewer’ role to support consistency and redistribute individual workloads. Administrative support, such as monitoring time intervals and creating clear, timely email templates for follow-up, helped to improve these metrics in the latter part of our study period.

We perceived that staff frustration with the process decreased as follow-up data began to show real impact. Finding the perfect balance between a personalised site-specific reporting timeline and a rigorous, standardised research reporting timeline remains a challenge. Therefore, we will continue our iterative process improvement cycle and aim to measure and report on the challenges and impact of our efforts prospectively.

### A synergistic approach to measuring the impact of a simulation programme

Our service sought a process that was synergistic with our mission and educational processes. Keeping our mission, ‘ensuring every child in Queensland has access to optimal resuscitative care’ forefront ensured we designed a process that supported our work rather than distracting from it.

At the practical level, we designed the summaries to serve multiple purposes. We use our site summaries to understand the impact of our service, but we use the same summaries in statewide meetings on recurrent themes throughout Queensland. In parallel, external staff members use them to advocate for changes in local steering groups, committees, and in individual emails to key stakeholders. Several regional educators have stated the process provided them with the external authority needed to push for movement on a disregarded issue.

The reporting process was a catalyst for change itself, providing us with the infrastructure needed to achieve what we had previously only hoped was happening. Our experience mirrors the conclusions of a systematic review of latent safety threats (LSTs) identified through in situ simulation, which highlight that LSTs are frequently identified but infrequently closed. The authors emphasise that ‘clear and structured processes need to be implemented for the resolution of patient safety threats identified through simulation’ [19].

The reporting system also helped us recognise issues that were shared across statewide hospital resuscitation systems, allowing us to advocate at higher levels for broader interventions. Our prior publication is now informing the design of a statewide paediatric drug safety profile, and adjustments to equipment ordering databases to ensure small hospitals can order small volumes of critical paediatric equipment.

We have recognised that the ‘transformational conversations’ we were having in our debriefs are more likely to impact the healthcare system when combined with diligent follow-up. We believe more simulation services could benefit from deliberately connecting findings from their learning conversations into quality improvement processes.

## Lessons learned and future avenues

To optimise translation of our learning to other services, we have synthesised five key lessons learned.

1. *Education is insufficient, but vital*
  Until we changed our strategy, the systemic challenges we uncovered did not improve. However, our prior decade of educational efforts were not wasted. Positive participant experiences and longitudinal relationships created the psychological safety needed to have harder conversations about the system. Simultaneously, our educators developed an incredible mental map of the issues faced by individual hospitals region-wide that heavily informed our understanding of statewide resuscitation.
2. *Iterative approaches were important*
  We received feedback from local educators requesting more direct communication with hospital stakeholders to support their efforts. We added additional contact lists to our data collection, carbon copied hospital executives, and reached out directly to stakeholders to support participants’ initiative when needed.
3. *The mission is what matters*
  While demonstrating impact on patient outcomes statewide was an alluring dream, it was not feasible and could have distracted us from more targeted, useful questions. Keeping our mission at the centre of all design choices, ‘i.e. how does this help sick kids’, ensured a streamlined approach with a meaningful outcome.
4. *Diligence is key*
  It was not an expensive new mannequin, a technological leap or a masterful debrief that magnified our impact; it was diligence. We postulate that simulation educators spend an extraordinary amount of time reflecting on the quality of their debriefs, but less time reflecting on how to harness those conversations for good, and how to connect that information to the right people. Our fullest potential was unlocked by diligent documentation, deliberate connection to key stakeholders, and gently belligerent follow-up.
5. *Local buy-in is essential*
  While our PRIME Site Summary provided important data to hospitals, it was local change agents who actioned every response. Sites with passionate nurse educators, pharmacists, and doctors were able to utilise the reports to build momentum for change. In one site in particular, we were humbled by the achievements of one nurse educator in closing every issue we found on every course. The reports and communication are catalysts for change, but the real heavy lifting is done by regional clinicians. This potential was hampered in sites with rapid staff turnover, but changes were remarkably dynamic in sites with long-term, experienced educators.
  It is our honest reflection that there are few things more transformative for a regional hospital than a passionate local clinician armed with unwavering conviction, an external report, and their patient’s best interests at heart. As one regional nurse educator put it, ‘This is our community. These are our kids. They deserve the best’ (L. Moore, personal communication, May 15th 2024).

## Limitations

Within this paper we share findings from 12 months of our new site reporting process. We have documented action on identified latent safety threats within all hospitals involved, but acknowledge some latent safety threats would have been closed whether or not we had flagged them. For example, as some sites roll out electronic medical records, more computers in resuscitation spaces are routinely installed, conveniently addressing our concerns about internet access in resuscitation rooms. Regardless of this, we are confident that site summaries played a significant role in many improvement processes, as evidenced by the numerous verbal and written communication channels we’ve been involved in since.

Identified latent safety threats are also specific to the curriculum of our course and shaped by the location of the simulation (in situ versus education rooms), equipment used (training drug infusion pumps versus actual ward equipment) and the perspectives of individual participants and faculty. We argue, however, that our goal was not to provide an objective measure of performance but to link valuable data generated in educational settings with organisational improvement systems. We have demonstrated such a system is both feasible and helpful for workplace improvement.

While we are confident our intervention impacted paediatric resuscitation systems, we have not proved improvement of actual paediatric resuscitations. Some of our recommendations may have unpredicted negative impacts; however, we argue this highlights the need for continuous cycles of performance improvement.

Our reporting system is context specific to Queensland hospitals and the design of our outreach courses; however, we publish our work not to suggest identical replication, rather to prompt simulation services to consider how they can embed measurement and advocacy more explicitly within their delivery.

## Conclusion

Simulation services seeking to measure impact need processes that are robust, achievable, and synergistic with their mission. We have described our simulation outreach model and presented the results of our robust reporting and advocacy process. Our use of software automation, administrative support, and longitudinal relationships made the process achievable without additional expense. We kept our processes synergistic with our mission by incorporating quality improvement methodologies into our existing educational courses, and in doing so have documented system problems and demonstrated tangible changes within healthcare systems at both the learner and institutional levels.

We hope this article provides detailed methods and generalisable principles for other simulation programmes seeking to demonstrate concrete outcomes, mitigate latent safety threats, and prove their value.

## Supplementary Information

Supplementary Material 1. Optimus PRIME site summary.

## Data Availability

No datasets were generated or analysed during the current study.

## Abbreviations

STORK: Simulation Training Optimising Resuscitation for Kids, the simulation outreach service for Children’s Health Queensland
PRIME: Preparing for Retrieval in Medical Emergencies, a course frequently delivered by STORK
ED: Emergency department
LSTs: Latent safety threats
