# Supplementary material for: Outreach simulation for system improvement: a novel advocacy and reporting process
Source: Adv Simul (Lond). 2025 Sep 1;10:44. doi: 10.1186/s41077-025-00372-0 (PMC12403477; doi:10.1186/s41077-025-00372-0)
Supplement: Supplementary file 1 — Supplementary Material 1. Optimus PRIME site summary. [file 41077_2025_372_MOESM1_ESM.pdf]

Optimus PRIME Site Summary:

Hospital Name

Date of visit: 29/07/2024 Report Ref: 88

Children's Health Queensland Optimus PRIME course aids teams throughout Queensland to optimise their team, environment and system for paediatric retrieval in medical emergencies. Today we considered DKA, Status Epilepticus, Emergency Intubation and Septic Shock management.

The following strengths, latent safety threats and ideas were identified by participants on the course. These issues are provided in good faith to assist local sites advocate for change and are not intended as an accreditation or auditing document.

Our team noted the following STRENGTHS of the service:	
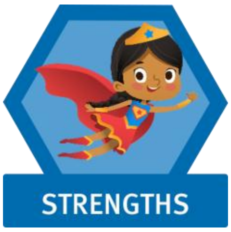	Staff reported pride for their service and the supportive relationships between local doctors and nursing staff. There were many positive reflections about the benefits of long term relationships built in a country setting, and how local GPs felt supported on call by their colleagues if needed in a paediatric emergency.<br>A new in situ simulation program is running monthly in the emergency department, with a mix of adult and paediatric cases. Staff reported this has been very helpful education, and built confidence in managing emergencies in general.

Issues with DRUG preparation, prescription and administration:	
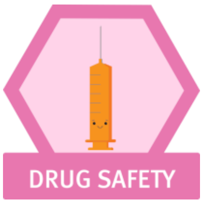	Phenytoin (20mg/kg over 20 mins) and Levetiracetam (60mg/kg over 5 minutes) could not be administered at the rate and dose currently recommended by Children's Health Queensland algorithms. Staff had to manually over-ride the software to administer Phenytoin at less than 60 minutes.
Suggested Actions:	Liaise with pharmacy regarding pump profile updates<br>Action Status: ACTION NEEDED
Contact Person:	regionalpharmacist@health.qld.gov.au

Issues with EQUIPMENT or LAYOUT of department:	
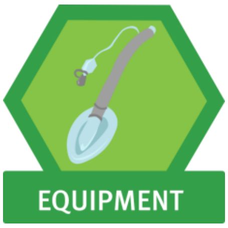	A new paediatric resuscitation trolley had recently been purchased, but stock for intubation was limited. Neonatal HME filters and neonatal nasopharyngeal airways were not available, and would be helpful in the event of a neonatal resus.
Suggested Actions:	Resus committee to consider purchasing neonatal HME filters and NPAs (see appendix for ordering codes)<br>Action Status: ACTION NEEDED
Contact Person:	localnurseeducator@health.qld.gov.au

Barriers to accessing appropriate PROTOCOLS/ALGORITHMS/RESOURCES:

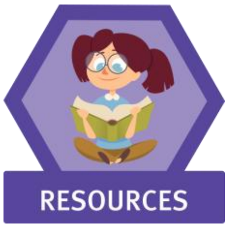	Staff had no access to a computer in their resus bay, and instead relied upon a manilla folder containing print outs of common paediatric algorithms. Several were from previous file versions or prior publications and were significantly out of date.<br><br>Access to paediatric resuscitation algorithms could be vital in an emergency, as such we suggest installation of a computer in resus or a version control process for printed algorithms.
Suggested Actions:	Action Status: ACTION NEEDED
Contact Person:	localemergencyphysician@health.qld.gov.au

Issues identified with SYSTEMS, PATHWAYS or TEAM RELATIONSHIPS:

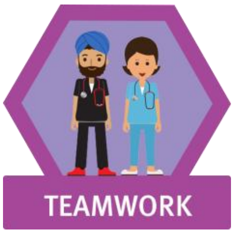	Staff recognised that there were many supportive relationships within the hospital, but that paediatric intubations on the ward could become challenging. In particular, a lack of clarity regarding the safest place to intubate (theatre vs the ward vs emergency) and who should attend a medical emergency response call (anaesthetics vs paediatrics vs emergency) contributed to the confusion.<br><br>Clarity regarding the medical esclation process in airway emergencies on the ward may be warranted.
Suggested Actions:	Resus committee assess current policy<br><br>Action Status: ACTION NEEDED
Contact Person:	localemergencyphysician@health.qld.gov.au

In addition to the above, STORK believes your site may wish to consider the following resources:

**Equipment codes:**	IV Bag Spikes;Mothership 2-way connectors (Fluid Dispensing Connector);
**Cognitive Aids:**	Access to QPEC Guidelines;
**Teaching Resources:**	Open access simulation packs for use in inter-departmental simulations: https://www.childrens.health.qld.gov.au/chq/health-professionals/qld-paediatric-emergency-care/education/optimus-bonus/
**Additional Courses:**	Optimus PULSE;

For further details, please contact your STORK Simulation Co-ordinator via stork.planning@health.qld.gov.au

Appendix 1. Frequently Requested Resuscitation Aides

Resuscitation Role Stickers

Ordered from Mediprint.com.au via email: sales@mediprint.com.au

Role	Code	Description
**DOCTOR**	**MC_LCCH007**	Emergency Label - Doctor
**AIRWAY DOCTOR**	**MC_LCCH009**	Emergency Label - Airway Doctor
**CIRCULATION DOCTOR**	**MC_LCCH002**	Emergency Label – Circulation Doctor
**PROCEDURE DOCTOR**	**MC_LCCH003**	Emergency Label – Procedure Doctor
**SURGICAL DOCTOR**	**MC_LCCH013**	Emergency Label – Surgical Doctor
**MEDICAL TEAM LEADER**	**MC_LCCH005**	Emergency Label – Medical Team Leader
**AIRWAY NURSE**	**MC_LCCH008**	Emergency Label – Airway Nurse
**CIRCULATION NURSE**	**MC_LCCH001**	Emergency Label – Circulation Nurse
**DRUG NURSE**	**MC_LCCH006**	Emergency Label – Drug Nurse
**NURSING TEAM LEADER**	**MC_LCCH010**	Emergency Label – Nursing Team Leader
**PROCEDURE NURSE**	**MC_LCCH004**	Emergency Label – Procedure Nurse
**NURSE**	**MC_LCCH011**	Emergency Label – Nurse
**SOCIAL WORKER**	**MC_LCCH015**	Emergency Label – Social Worker
**XRAY**	**MC_LCCH014**	Emergency Label – X-Ray
**CPR COACH**	**MC_LCCH030**	Emergency Label – CPR Coach

Item	Description	Company	Order Code
**FLUID DISPENSING CONNECTOR**	CONNECTOR:FLUID:DISPENSING:BOX/100	S/4 HANA	10033158
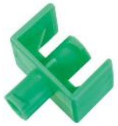	1:1 Syringe Connector for preparation of resuscitation drugs utilising the 'Mothership method' from CREDD book.<br>Allows decanting of individual resuscitation doses of medication from a from a larger standardised concentration dose.		
**PHENYTOIN FILTER**	FILTER:SYRINGE:MINISART:0.2M:SARTORIUS	S/4 HANA	10032058
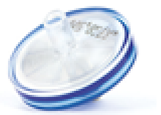	0.22 micron filter for phenytoin infusion.<br>Phenytoin is insoluble in water and can precipitate when mixed with other drugs or diluents.<br>The use of a 0.22-micron filter helps remove any particles or debris ensuring that the medication is delivered safely and effectively.		
**CREDD book**		Children's Health Queensland	
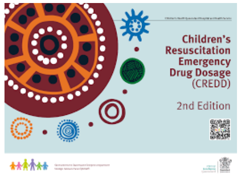	Standardised drug preparation guide for paediatric resuscitation and equipment sizing.	Available electronically free of charge via QPEC	To order physical copies please email stork@health.qld.gov.au
**BAG SPIKES**	Bag spike with smartsite, length 8.3cm, priming volume 0.47ml, DEHP free	S/4 HANA	10063289
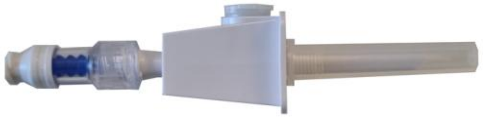	Allows for needle free withdrawal of multiple doses of fluid when preparing drugs		
**Pre-Filled Medication Labels**	Pre-Filled Labels	QHealth intranet policy	
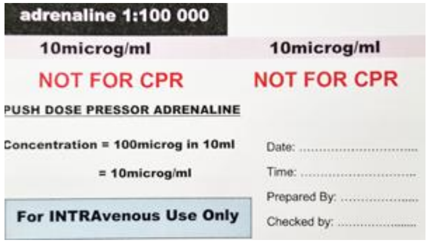	Drug specific medication labels for increased legibility and consistency with paediatric drug preparation.<br>Paediatric drug preparation is a recognised source of increased cognitive load for resuscitation teams.	User-applied labelling requirements at QCH including governance of locally developed labels	Sites need to contact their Pharmacy for approval, who then need to contact the CHQ Medication Safety Committee CHQMedicationSafety@health.qld.gov.au

Item	Description	Company	Order Code
**PROV-ED Airway Dropsheet**	SSIP Pack	PROV-ED	
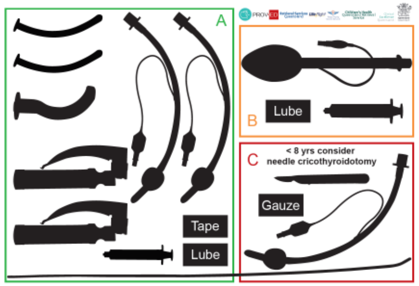	An intubation drop sheet designed to standardise and simplify intubation tray preparation<br><br>This dropsheet comes as a component of the PROV-ED 'Standardised and Safe Intubation Package'.		Email: PROVED.Project@health.qld.gov.au<br><br>To request an SSIP (Standardised & Safe Intubation Package) be delivered to your service.
**Team Brief Checklist**		STORK	
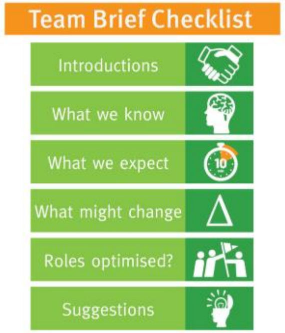	An effective team brief positively impacts team preparation and resuscitation.<br><br>Based on publication by Purdy et al, this team briefing checklist aims to help standardise team briefings in the emergency department.	Available for free download via the following links:<br>- [A3 poster]<br>- [A4 sheet]	
**Paediatric Lanyard Cards**		Paediatric Essential Cards	
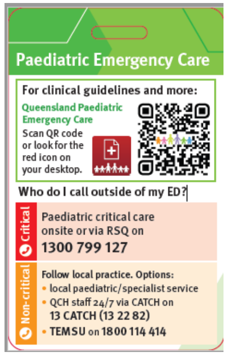 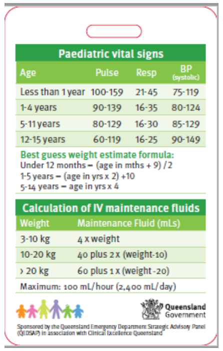	Provide normal range for different age groups, fluid calculation and best weight estimates and QR code links to clinical guidelines and retrieval contacts.		Email: Sally Smith<br>Sally.smith@mcdonald.group

Paediatric Nasopharyngeal Airway		S4/HANA
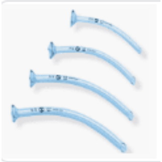	Size 3.0 AIRWAY:NASOPHARYNGEAL:ST:SU:4.7MM<br>Size 3.5 AIRWAY:NASOPHARYNGEAL:ST:SU:5.3MM<br>Size 4.0 AIRWAY:NASOPHARYNGEAL:ST:SU:6.0MM	10033825<br>10016928<br>10033826
**ENFIT Nasogastric Tube**		**S4/HANA**
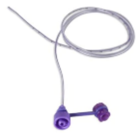	TUBE:FEED:NASOG:8FX80CM:SHT TERM:ENFIT/ Box 50<br><br>8FR Short-term Nasogastric Tube. Used for gastric decompression and feeding	10052442
**ENFIT Syringe**		**S4/HANA**
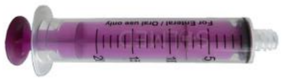	SYRINGE:FEED:ENTERAL:ENFIT:SU:20ML<br><br>20ml Purple Enteral Syringe to fit ENFIT NGT	10051693
**Small disposable manometers**		**S4/HANA**
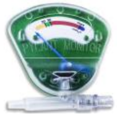	Disposable manometer to measure ETT cuff pressure post intubation	10012105
**Paediatric HME Filters**		**S4/HANA**
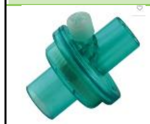	Decreased dead-space with built in ETT connection point<br>Neonatal HME 30-100mL TV<br>Paediatric HME 75-300mL TV	 10033517<br>10061939
**Paediatric High Flow Nasal Prongs**		**S4/HANA**
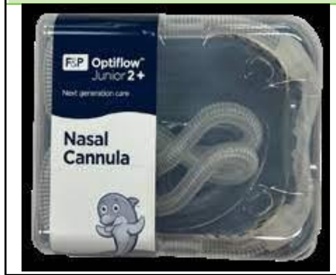	Dolphin XXL Optiflow paed nasal prongs for High flow 10-50L flow	10060088
